# Deviations of personality development in children from families of different social status

**DOI:** 10.1192/j.eurpsy.2021.1192

**Published:** 2021-08-13

**Authors:** N. Burlakova, P. Davidovich

**Affiliations:** 1 Neuro- And Pathopsychology, Lomonosov Moscow State University, Moscow, Russian Federation; 2 Department Of Psychotherapy, Pirogov Russian National Research Medical University, Moscow, Russian Federation

**Keywords:** social status, Personality, genetic approach, deviations of personality development

## Abstract

**Introduction:**

Methodological foundations of Lev Vygotsky’s school enable to reveal the mechanisms shaping personality disorders and to explore the process of disorder formation. The genesis of symptoms is examined in the context of the social situation of child’s development and historical environment.

**Objectives:**

2 groups of children (aged 5,5-7): (1) upper middle class, from prestigious development center (n = 31); (2) lower middle class, from social assistance center (n = 35).

**Methods:**

The following methods were used: СAT (Bellak); objective description of the cultural and social context of the child’s development; long-term observation.

**Results:**

1) Deviations of personal development in children from the first group are expressed in individualism and related narcissism. This feature is actualized in CAT stories with the situation of rivalry between the characters (t. 1, 4, 5): the central character is either ignored by the child, or pampers the “small” character, which represents the child’s identification model (t. 3, 7). Another feature is intolerance to frustration; frustration is ignored in pictures dealing with this topic (t. 8, 10). 2) For the second group, typical features include anxiety, rigidity when acting not according to the rules. These features are manifested at the beginning of the CAT test and in situations when self-expression is required (t. 1). Moreover, children demonstrate the negative sense of self, which is expressed in identification with losing characters (t. 2) and in projections of the early social fears (t.8)

**Conclusions:**

The social and genetic approach enables more thorough and careful examination of the onset of the deviation.

